# Cases of Vesico-Vaginal Fistula

**Published:** 1862-12

**Authors:** De Laskie Miller

**Affiliations:** Prof. of Obstetrics in Rush Medical College


### CHICAGO MEDICAL JOURNAL.

VOL. VI.] DECEMBER, 1862. [No. 12.

### ORIGINAL COMMUNICATIONS.

### CASES OF VESICO-VAGINAL FISTULA.

By DE LASKIE MILLER, M. D.,

*Prof. of Obstetrics in Rush Medical College.*

CASE 1ST.—Vesico-Vaginal Fistula—Following Protracted Labor—Spontaneous recovery.

Mrs. H., aged about 33 years, of large frame and strong muscular development, had about three years previously given birth to a still-born child, after a tedious labor. Was taken with labor pains about 2 o'clock A. M. Saturday, and by direction of the midwife in attendance, she exerted herself to the utmost, from the first pains, to increase, by voluntary efforts, the efficiency of the uterine contractions, till Monday evening following, when from physical exhaustion the progress of labor was suspended.

At this time, by a vaginal examination, I found the os uteri dilated; the head engaged in the superior strait, in the first position; the vagina hot, dry and tender; the urine had not been voided for many hours The distended bladder was relieved by the catheter. The forceps were applied without great difficulty, and after a little while the child was delivered still-born.

At first the only unfavorable symptom was a partial paralysis of motion and sensation in the right inferior extremity. The bladder was evacuated by the catheter, and the bowels moved with Ol. Ricini on the second day. The lochia continued in the usual quantity, but offensive.

On the 8th day after delivery the patient complained of inability to retain her urine, and an examination, made by passing a silver catheter into the bladder and a finger into the vagina, revealed a fistula nearly the size of a ten cent piece, opening into the bladder at the upper end of the trigone.

*Treatment.*—The bowels were kept soluble by the use of saline laxatives. The catheter was allowed to remain in the bladder a considerable portion of the time. The vagina was syringed frequently with tepid water. Electro-magnetism and stimulating frictions were applied to the paralysed limb. After about two weeks the fistula appeared to have contracted sensibly; sensation and power began to return in the limb; and in about six weeks more the urine passed only by the urethra, and the patient was enabled to walk without assistance. The improvement in the limb continued until the patient perfectly recovered.

The result in this case impressed me most forcibly with the importance of assiduous attention, in recent cases of accidents of this kind, and of the probability of recovery by the constant use of a properly constructed catheter, and frequent use of vaginal and vesical injections of water, or with the addition of some suitable astringent.

Case 2nd.—Mrs. A., from an adjoining State, the mother of two children—the youngest about seven years of age—arrived in this city Sept. 20th.

The history of her case, as obtained from herself, was, briefly: That about five weeks previously she was taken in labor; that after suffering about seven days under the care of her physician, who had administered ergot freely, she secured the services of another physician, who delivered her, with instruments, of a large still-born child. A few days after her delivery, she experienced inability to retain her urine, which flowed continuously. The physician last in attendance examined and found a vesico-vaginal fistula, situated high up in the vagina; and by his advice the patient visited this city to obtain relief.

Having evacuated the bowels freely with Ol. Ricini on Sunday, I performed the operation Monday A. M., assisted by Profs. Brainard and Ingals, Drs. Lynn and Gore, and Mr. G. B. Cloud, medical student. The patient was placed in the prone position, leaning over the edge of a table, the hips elevated above the shoulders by pillows properly placed, and chloroform administered. The edges of the fistula were then carefully dissected so as to denude the entire thickness from the vaginal to the vesical mucous membrane. Three stitches of silver wire were then introduced, with the precaution of not including any portion of the vesical mucous membrane. No little difficulty was experienced in passing the needles through the upper border, for the fistula encroached very closely upon the neck of the uterus. The shield was then brought into requisition, it having been previously prepared from thin sheet lead, of a size somewhat larger than the fistula and oval in shape, measuring about $1\frac{1}{4}$ inches by $\frac{3}{4}$ of an inch, and perforated with six small holes, about $\frac{1}{4}$ of an inch apart, and arranged in two rows, separated about 1-16 of an inch. The wires were then passed through these holes while the plate was still external to the vulva. The plate was then carried up to the fistula and sufficient traction made to bring the edges together. The wires were then twisted close to the shield and cut off. A catheter was introduced into the bladder, the patient placed on her back in bed, and 2 grs. of Pulv. Opium administered. This was repeated three times in the succeeding thirty-six hours. The diet consisted principally of nutritious broths and beef tea. The vagina and bladder were washed by daily injections of cool water. Very little pain or irritation of any kind followed the operation. The bowels were not evacuated until the eighth day.

On the 9th day the stitches were taken out and the shield removed. The fistula was completely closed. The catheter was retained in the bladder several days longer, and the patient remained about a week after discontinuing the use of the instrument, and then returned to her home perfectly well.

CASE 3RD. Still under treatment.—Mrs. C. was delivered of her first child about six months since. After suffering in labor more than a week, instruments were used, and the child was still-born. By her account the fistula existed from the time of delivery, for she represents that she was unable to control the escape of urine thereafter.

The operation, which I performed one week since, did not differ in any essential particular from that described in the second case. No unfavorable symptom has occurred. The shield has not yet been removed, but, from appearances, the case promises to terminate favorably.

